# Experimental Infection of *Rhodnius prolixus* (Hemiptera, Triatominae) with *Mycobacterium leprae* Indicates Potential for Leprosy Transmission

**DOI:** 10.1371/journal.pone.0156037

**Published:** 2016-05-20

**Authors:** Arthur da Silva Neumann, Felipe de Almeida Dias, Jéssica da Silva Ferreira, Amanda Nogueira Brum Fontes, Patricia Sammarco Rosa, Rafael Enrique Macedo, José Henrique Oliveira, Raquel Lima de Figueiredo Teixeira, Maria Cristina Vidal Pessolani, Milton Ozório Moraes, Philip Noel Suffys, Pedro L. Oliveira, Marcos Henrique Ferreira Sorgine, Flavio Alves Lara

**Affiliations:** 1 Laboratório de Microbiologia Celular, Oswaldo Cruz Institute, Oswaldo Cruz Foundation, Rio de Janeiro, Brazil; 2 Laboratório de Bioquímica de Artrópodes Hematófagos, Federal University of Rio de Janeiro, Rio de Janiero, Brazil; 3 Laboratório de Biologia Molecular Aplicada a Micobactérias, Oswaldo Cruz Institute, Oswaldo Cruz Foundation, Rio de Janeiro, Brazil; 4 Lauro de Sousa Lima Institute, Department of Biology, Bauru, Brazil; 5 Laboratório de Hanseníase, Oswaldo Cruz Institute, Oswaldo Cruz Foundation, Rio de Janeiro, Brazil; Instituto Nacional de Salud Pública, MEXICO

## Abstract

Leprosy is a chronic dermato-neurological disease caused by infection with *Mycobacterium leprae*. In 2013 almost 200,000 new cases of leprosy were detected around the world. Since the first symptoms take from years to decades to appear, the total number of asymptomatic patients is impossible to predict. Although leprosy is one of the oldest records of human disease, the mechanisms involved with its transmission and epidemiology are still not completely understood. In the present work, we experimentally investigated the hypothesis that the mosquitoes *Aedes aegypti* and *Culex quinquefasciatus* and the hemiptera *Rhodnius prolixus* act as leprosy vectors. By means of real-time PCR quantification of *M*. *leprae* 16SrRNA, we found that *M*. *leprae* remained viable inside the digestive tract of *Rhodnius prolixus* for 20 days after oral infection. In contrast, in the gut of both mosquito species tested, we were not able to detect *M*. *leprae* RNA after a similar period of time. Inside the kissing bug *Rhodnius prolixus* digestive tract, *M*. *leprae* was initially restricted to the anterior midgut, but gradually moved towards the hindgut, in a time course reminiscent of the life cycle of *Trypanosoma cruzi*, a well-known pathogen transmitted by this insect. The maintenance of *M*. *leprae* infectivity inside the digestive tract of this kissing bug is further supported by successful mice footpad inoculation with feces collected 20 days after infection. We conclude that *Rhodnius prolixus* defecate infective *M*. *leprae*, justifying the evaluation of the presence of *M*. *leprae* among sylvatic and domestic kissing bugs in countries endemic for leprosy.

## Introduction

According to WHO, the global registered prevalence of leprosy in 2013 stood at 189,018 new cases around the world, the large majority in tropical countries [1]. Despite these numbers, leprosy is not a tropical disease *stricto senso*, since it was highly prevalent in Europe until the 19^th^ century. Being the first reported and well documented disease in the history of medicine, leprosy was described in China, India and Egypt already in 600 A.D., being the first disease related to an etiological agent, *Mycobacterium leprae*.

The most accepted transmission route of leprosy is through prolonged contact with multibacillary patients, the main bacilli shedders. Due to host genetics, nutrition and immunological factors, it is presumed that only 3–5% of human population presents the genetic and immunologic susceptibility to develop leprosy [2]. The theory that recognized multibacillary patients as the only source of infection does not explain how Europe virtually eradicated leprosy one century before rifampicin use, or why developed countries do not observe autochthonous cases of the disease, even receiving massive migration from highly endemic countries. Most important, although the reported number of multibacillary patients under treatment has drastically declined worldwide, the trend in decline stabilized several years ago [1, 3, 4].

Although being a strictly intracellular parasite, a number of data indicate that *M*. *leprae*, similar to other uncultivable mycobacteria, could be maintained alive in environment samples such as water reservoir and soil or inside amoebas [5–7]. In 1975, the first report of naturally acquired leprosy in armadillos (*Dasypus novemcinctus*) was published [8], but the idea of leprosy as a sporadic zoonosis took about four decades to be accepted. In 1983 it was confirmed that the acid-fast bacilli isolated from wild armadillos were indeed *M*. *leprae* [9] and since then, numerous surveys have confirmed that armadillos in the southern United States are a natural reservoir for *M*. *leprae*, with natural populations presenting a prevalence around 20% [10, 11]. Recently, by means of genome sequencing, Truman and colleagues demonstrated that armadillos and humans were infected with the same *M*. *leprae* strain, strongly indicating interspecies transmission [12]. The idea that leprosy transmission could be sustained by a mammalian reservoir in nature such as armadillos and monkeys could explain why some countries, such as Brazil, continue to register constant rate of new cases of leprosy along decades, in spite of the reduction of poverty and improved income distribution. Most important, disease incidence persisted even when there was progressive drop of multibacillary patients among population due to WHO polychemotherapy use [3, 13].

Leprosy transmission by insects is an old hypothesis [14, 15], which could explain how the bacteria circulate from armadillos to humans in US and Brazil, where the disease is highly prevalent at the agricultural frontier regions of the most depopulated states in the amazon region with an average of 6 cases / 10,000 habitants, and regarding the big cities large poverty populations at south east of 1 case / 10,000 habitant [16]. Some authors described experimentally leprosy transmission by *Aedes aegypti*, *Culex fatigans* and flies [14, 17–22]. Those studies, however, were limited by the tools available at that time to study a non-cultivating mycobacteria: acid-fast staining combined with fluorescence, light and electronic microscopy. Due to the fact that insects are naturally colonized by acid-fast mycobacteria, their observation in the midgut of insects fed on multibacillary patients were not accepted as demonstration of infection.

Armadillos are recognized as mammals that are parasitized by kissing bugs, the latter being reported as a reservoir not only of *M*. *leprae*, but also of *Trypanosoma cruzi*, the etiological agent of Chagas Disease [23]. It is well known that kissing bugs are frequently found in *Dasypus novemcinctus* armadillo burrows, feeding on their blood [24]. In nature, up to 75% of the armadillo diet is based on insects, affording ample opportunity for infection through ingestion of kissing bugs [25]. Although an oral route of infection has been described in Chagas Disease and related to a more acute form of disease [26, 27], this way of pathogen entry is still controversial in the case of leprosy [28].

In the present work, we hypothesize that kissing bugs such as *Rhodnius prolixus*, an insect from the family Reduviidae within the order Hemiptera (infra-order: Heteroptera), known as vectors of Chagas Disease, could also be a vector of Leprosy. We used immunofluorescence and real-time PCR of 16SrRNA to localize and quantify *M*. *leprae* Thai-53 strain along the digestive tract of the mosquitoes *A*. *aegypti* and *C*. *quinquefasciatus* and of the kissing bug *R*. *prolixus*. In contrast with mosquitoes, *R*. *prolixus* is able to maintain *M*. *leprae* viability inside its digestive tract for at least 20 days. This period is enough for the insect to start a new blood meal, defecating a large amount of live infectious bacilli. Studies are needed to evaluate the contribution of naturally infected kissing bugs to the transmission of leprosy.

## Material and Methods

### Insect infection

*Rhodnius prolixus*, *A*. *aegypti and C*. *quinquefasciatus* were maintained in a biosafety insectary and fed with rabbit blood containing 10^7^/mL live *M*. *leprae* Thai-53 strain through a latex membrane (*R*. *prolixus*) or a plastic paraffin film (*A*. *aegypti* and *C*. *quinquefasciatus*) coupled to a glass chamber and maintained at 37°C with the use of a circulating water bath. The insects were allowed to feed in the dark for no more than one hour, a time interval that did not reduce *M*. *leprae* viability. After this time, insects not fully engorged were discarded. The adult kissing bugs and mosquitoes typically ingested blood meal volumes of about 250μl and 2μl respectively. After feeding, 13 groups of 5 *R*. *prolixus* each, and 10 groups of 10 *A*. *aegypti* or *C*. *quinquefasciatus*, were kept at 80% humidity and 27°C. Mosquitoes were allowed to feed *ad libitum* a 10% sucrose solution. All insects were kept in those conditions for 2 hours or 20 days after blood meal (ABM) until tissue dissection.

### *Mycobacterium leprae* preparation and viability determination

*Mycobacterium leprae* was purified from nude mouse footpad as described [29]. Briefly, both Foxn1^nu/nu^ mice hind footpad were inoculated with 10^4^ live *M*. *leprae*. Animals were monitored each 48 h during seven months. *M*. *leprae* infection in mice is recognized as painless, without the display of any symptoms besides footpad swelling, and for that reason analgesic protocol was not necessary. None of the animas died as a result of *M*. *leprae* infection. After seven months, animals were sacrificed through intraperitoneal administration of 15 mg/Kg and 150 mg/Kg of xylazine and ketamine (Vallée, SP, Brazil) respectively, followed by cervical dislocation. After skin and bones were removed, tissue was reduced in small pieces by scissors and digested with a solution of 170 units of colagenase type I, 2 units of dispase (Life Technologies, NY, USA), 50μ mg/mL of ampicillin (Sigma, St. Louis, USA) and 150 units of DNAse (Life Technologies, NY, USA) during 2 h at 33°C. Digested tissue was homogenized by vortex and washed, three times in water, one time in NaOH 0.1 N and one time in RPMI medium, by centrifugation at 10,000 g/5 min, and counted by acid-fast staining (Ziehl-Neelsen Kit, Becton Dickinson). For evaluation of the preparation viability, a fluorimetric staining protocol was used based on the LIVE/DEAD Bactlight Bacterial viability Kit (Life Technologies, CA, USA), performed according to the manufacturer’s instructions [30]. To optimize artificial infection yield, we only used bacterial preparations with a viability of at least 85%. Freeze-thaw inactivated *M*. *leprae* was used to evidence 16Sr RNA instability inside triatomines digestive tract. Before infection, live *M*. *leprae* was frozen in dry ice and thaw at 37°C water bath five times. Although freeze-thaw does not drastically alter RNA content compared to other inactivation techniques such as irradiation or heat, *M*. *leprae* inactivation by freezing is a well characterize phenomenon [31].

To determine viable *M*. *leprae* titer inside insects digestive tract we used qPCR 16S cDNA/DNA rate, as described elsewhere [32]. Briefly, a pool of 10 mosquitoes or *R*. *prolixus* two hours or 20 days after feeding on blood were dissected in PBS and mosquitos thorax and abdomen or *R*. *prolixus* posterior midgut, hindgut and rectum were removed under sterile conditions and transferred to FastRNA Blue tubes (MP Biomedicals), containing 1mL of TRIzol. Tissues were homogenized by vortex and *M*. *leprae* RNA and DNA were extracted after disruption in Fast Prep FP 24 homogenizer (MP Biomedicals, CA, USA) at a speed of 6.5 m/s for 45 sec. The tubes were cooled on ice for 2 min between two sections of disruption. After homogenization the tubes were cooled for 5 min and then received 200 μl of chloroform. After rapid mixing by inversion, tubes were centrifuged at 12,000 x g at 4°C for 15 min. The upper fase containing the RNA was transferred to a new tube, treated with DNA-Free kit (Ambion, Inc., Austin, USA) as specified by the manufacturer and precipitated by standard ethanol technique. Precipitated RNA was solubilized in RNAse free water and stored at -80°C until use. The DNA was purified by adding 100 μl of 10 mM Tris-EDTA (pH 8.0) and 150 μl of isoamyl alcohol and precipitated with 0.3 M sodium acetate with two volumes of cold ethanol. The DNA pellet was washed in 70% ethanol, dissolved in 30 μl of sterile distilled water and stored at -80°C until use. Qualitative RNA analysis was performed in agarose gel electrophoresis and quantification of both DNA and RNA were performed in NanoDrop One (Thermo Fisher Scientific, MA, USA). The *M*. *leprae* RNA was reverse transcribed using random primers and superscript III following manufacturer’s instructions (Invitrogen, CA, USA). The levels of *M*. *leprae* 16SrRNA mRNA and DNA were determined in all tissues by real-time RT-PCR, using the primer pairs sense 5’ GCA TGT CTT GTG GTG GAA AGC ‘3 and anti-sense 5’ CAC CCC ACC AAC AAG CTG AT ‘3. The PCR reaction mixes were 50°C for 2 min and 95°C for 10 min, followed by 40 cycles of 95°C for 15 sec and 60°C for 1 min monitoring SYBR Green fluorescence in an ABI StepOne Plus System (Applied Biosystems, CA, USA). In order to convert RNA/DNA Real-Time PCR signal in number of live *M*. *leprae* genomes, we added different concentrations of live *M*. *leprae*, ranging from 10^8^ to 10^1^, in non infected insect tissues extracts dissected 2h and 20 days after blood meal, building fourteen curves, one for each condition. Their angular coefficient were used to determined the number of viable *M*. *leprae* in each condition.

### Fluorescence microscopy

In order to observe *M*. *leprae* in triatomine feces, we infected the insects with *M*. *leprae* pre-stained with PKH-26 fluorophore (Sigma-Aldrich, St. Louis, USA) according to the manufacturer instructions. In all other tissues, non-fluorescent *M*. *leprae* was evidenced by immunostaining as followed. After artificial feeding, the triatomines tissues were fixed with 4% paraformaldehyde for 24 h at 4°C and cryopreserved by a two hour sequential incubations in PBS containing 15% and 30% sucrose. Soon after, the tissues were embedded in O.C.T. (Sakura Finetechnical, Tokyo, Japan), immediately frozen in liquid nitrogen and 10 μm semi thin sections were prepared using a Leica CM3050 cryostat and mounted on KCr (SO_4_)_2_ gelatinized slides.

*M*. *leprae* immunolocalization was performed using the monoclonal Anti-*M*. *leprae* lipoarabinomannan (LAM) antibody CS-35, kindly provided by Biodefense and Emerging Infections Research Resources Repository at http://www.beiresources.org/TBVTRMResearchMaterials/tabid/1431/Default.aspx. Briefly, slides were permeabilized and blocked by 30 minutes incubation with 0.01% Triton X-100 and 10% of fetal bovine serum in PBS pH 7.2. Tissues were incubated for 2 hours with mouse IgG anti-LAM antibodies (1:50 vol/vol) and nuclei were stained by DAPI (Sigma-Aldrich, St. Louis, USA). Secondary antibodies conjugated with Alexa 633 IgG anti-mouse; (Invitrogen, CA, USA) were incubated with the samples for an additional 2 h and tissue observed in a Zeiss Axiobserver Z1 with a Colibri illumination system (Carl Zeiss, Heidenheim, Germany).

### *Rhodnius prolixus* feces infectivity determined using the Shepard’s model

In order to check triatomine feces for *M*. *leprae* infectivity, we used a model based on inoculation of *M*. *leprae* into mice footpads and counting acid-fast bacilli after 6 months, as previously described. Briefly, we infected *R*. *prolixus* with blood containing 10^7^/ml PKH-26 stained *M*. *leprae* according to manufacturer instructions, a procedure known as non-interfering with mycobacteria viability [33]. After 20 days, *R*. *prolixus* were fed with non-infected blood. Feces from 8 pools of 10 insects each were collected and pelleted by 10.000g / 5 min. The pellet was homogenized in a sterile solution of NaOH 0.1N during 5 minutes followed by centrifuging (10.000g / 5 min). The pellet was resuspended in sterile PBS and counted by fluorescence microscopy and Fite-Faraco Staining. Only samples which presented negative contaminants grow after 24h culture on blood agar and TH10 plates at 33 and 37°C (6 from 8 samples) were inoculated in animals. A total of 10^4^ *M*. *leprae* were injected per BALB/c hind foot pad (ILSL, São Paulo, Brazil), using a conventional Sheppard's infection model [34]. As positive control we inoculate 10^4^ *M*. *leprae* purified from nude mice foot pad. A negative control was generated by treating animals with 10mg/Kg/week rifampicin by weekly gavage during five months, one month after inoculum with 10^4^ Thai-53 *M*. *leprae* purified from nude mice foot pad.

Animals were monitored each 48 h during seven months. None animal died as a result of *M*. *leprae* infection. After six months, animals were sacrificed through intraperitoneal administration of 15 mg/Kg and 150 mg/Kg of xylazine and ketamine (Vallée, SP, Brazil) respectively, followed by cervical dislocation, and foot pads were excised and macerated for bacillary counting by Fite-Faraco Staining.

### Ethics Statement

Animal protocols were in agreement with the 8^th^ edition of the Guide of Care and Use of Laboratory Animals of the National Institutes of Health. All procedures were approved by the Animal Welfare Committee of Sagrado Coração University (São Paulo, Brazil), responsible for Instituto Lauro de Souza Lima animal care and use inspections, were all animal protocols were performed according license number 219/11.

## Results

**Fig 1:**
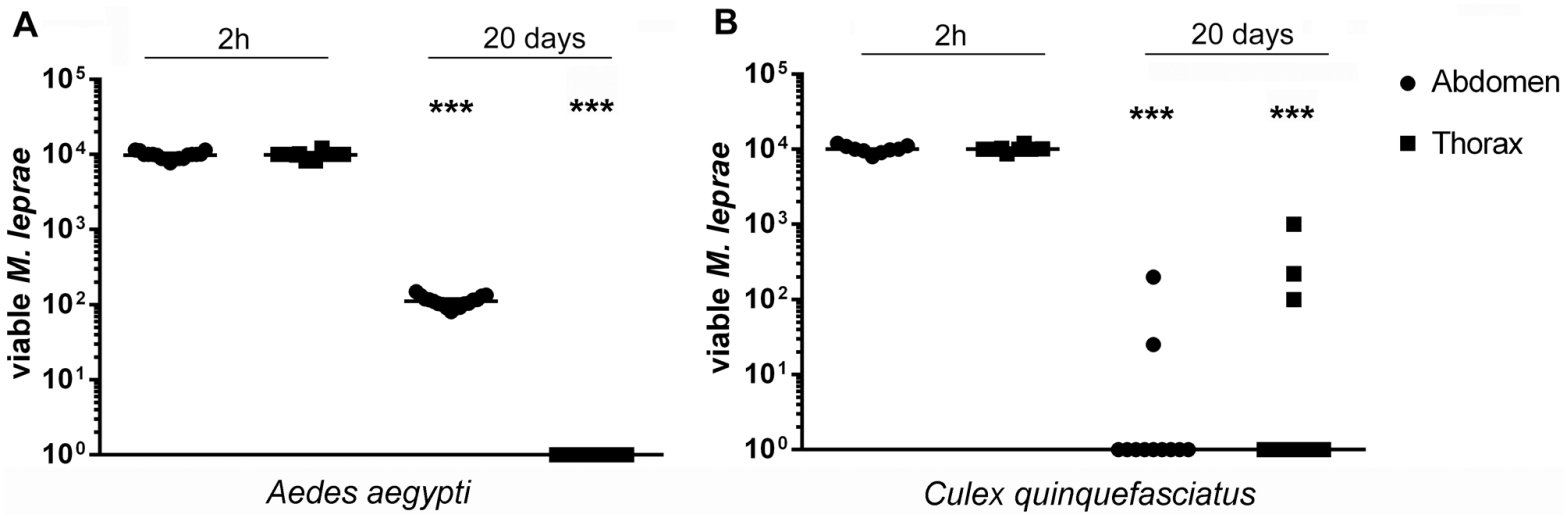
*M*. *leprae* loss viability in mosquitoes. The number of life *M*. *leprae* was determined by *M*. *leprae* 16SrRNA levels in *Aedes aegypti* (A) and *Culex quinquefasciatus* (B) abdomen (spheres) and thorax (squares). All mosquitoes were dissected 2 hours and 20 days after blood meal. Line represents median and each point is a pool of 10 mosquitoes, obtained in three independent experiments. Non-infected controls did not show amplification of the targets (CT>39).

There are several reports on mosquitoes capacity to carry *M*. *leprae* from one mammal to another through biting [18], being able to maintain its cell wall morphology inside their digestive tract for until four days [19], as the only viability parameter used on that moment. To verify if *M*. *leprae* maintains its viability after ingestion by *A*. *aegypti* or *C*. *quinquefasciatus* using a reliable molecular tool, these mosquitoes were offered a blood meal containing 10^7^ live *M*. *leprae* / blood mL, and 16SrRNA levels in the abdomen containing: posterior midgut, ventral diverticulum and hindgut; or thorax and head containing: proboscis, salivary glands and foregut; were measured by qPCR (Fig 1).

As we can see, viable *M*. *leprae* levels inside *Aedes aegypti* abdomen 20 days after blood meal (ABM) are at least two fold lower than levels observed immediately after feeding (2 hours ABM) (Fig 1A). *M*. *leprae* viability was virtually abolished inside *C*. *quinquefasciatus* digestive tract, presenting a very low positivity only in two and three groups of abdomen and thorax respectively (Fig 1B), indicating that neither *A*. *aegypti* nor *C*. *quinquefasciatus* presents, in our model, potential to transmit leprosy.

We concluded that there is a lack of an essential characteristic of a disease vector in both mosquito species regarding its interaction with *M leprae*, which is the ability to keep the pathogen infectious until the next blood meal.

**Fig 2:**
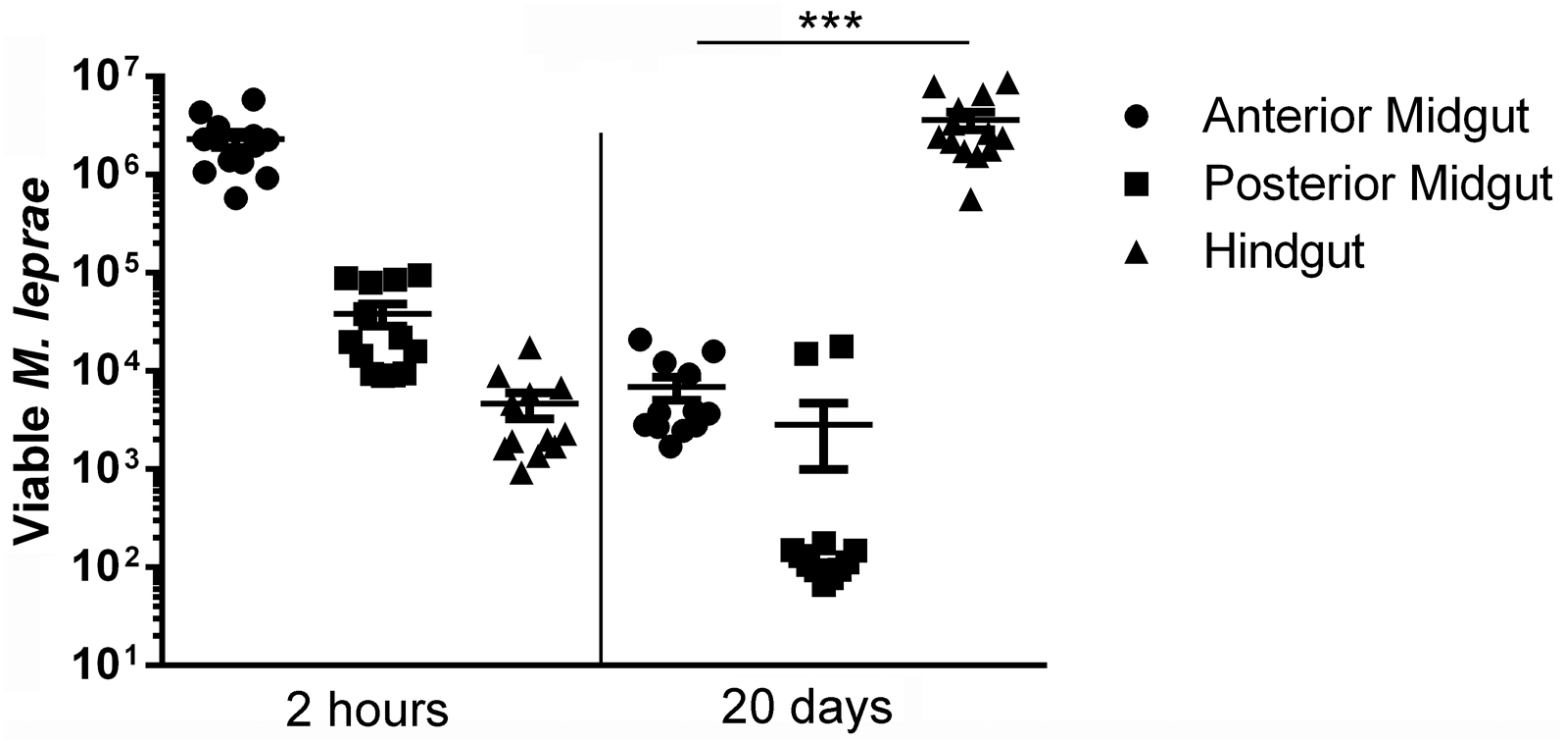
The kissing bug *Rhodnius prolixus* is able to maintain *M*. *leprae* viability inside its digestive tract after infection. *M*. *leprae* viability was determined by the persistence of 16Sr RNA at the different digestive compartments of artificially infected adult *Rhodnius prolixus*: anterior midgut (spheres), posterior midgut (squares) and hindgut (triangles), just after blood meal (2h) and after total blood meal digestion (20 days), infected with the pathogen. As we can see, the hindgut was the only compartment where the level of living *M*. *leprae* increases after 20 days of infection. Scatter plot showing mean and SEM of four independent experiments, each point represent five insects group. *** means p < 0.001 (2h x 20 days, for each group of sample). Non-infected controls did not present amplification of the targets.

Although there are some circumstantial evidence suggesting that armadillos could be infected by Culex mosquitoes with the St. Louis encephalitis virus in Florida (USA) [35], larger insects, such as kissing bugs, were more frequently found feeding on armadillos, which are recognized sylvatic reservoirs of Chagas Disease [24, 36], transmitted by several species of triatomine bugs in Central and South Americas [37]. *Rhodnius prolixus* has been used over the last decades as a model to study Chagas Disease vectoring [38], due to its adaptability to artificial feeding and growth in laboratory. For these reasons, our next approach was to measure the same parameters observed in mosquitoes, in the kissing bug digestive tract compartments: anterior midgut, posterior midgut and hindgut. When *Rhodnius prolixus* adults were fed with blood containing *M*. *leprae*, (Fig 2) high viability was detected in anterior midgut and hindgut of the insects even 20 days after infection. During this time interval, *M leprae* levels dropped in posterior midgut, but increased 100-fold in the hindgut, suggesting that the bacteria were moving along with the blood meal. Our data suggests that there was no mycobacterial replication along these 20 days of blood digestion, but the bacteria remained alive, due to the fact that insects exposed to *M*. *leprae* inactivated just before ingestion by freeze-thaw was not able to sustain detectable levels of 16Sr RNA in none of the insect digestive compartments after the same period of time (S1 Fig).

**Fig 3:**
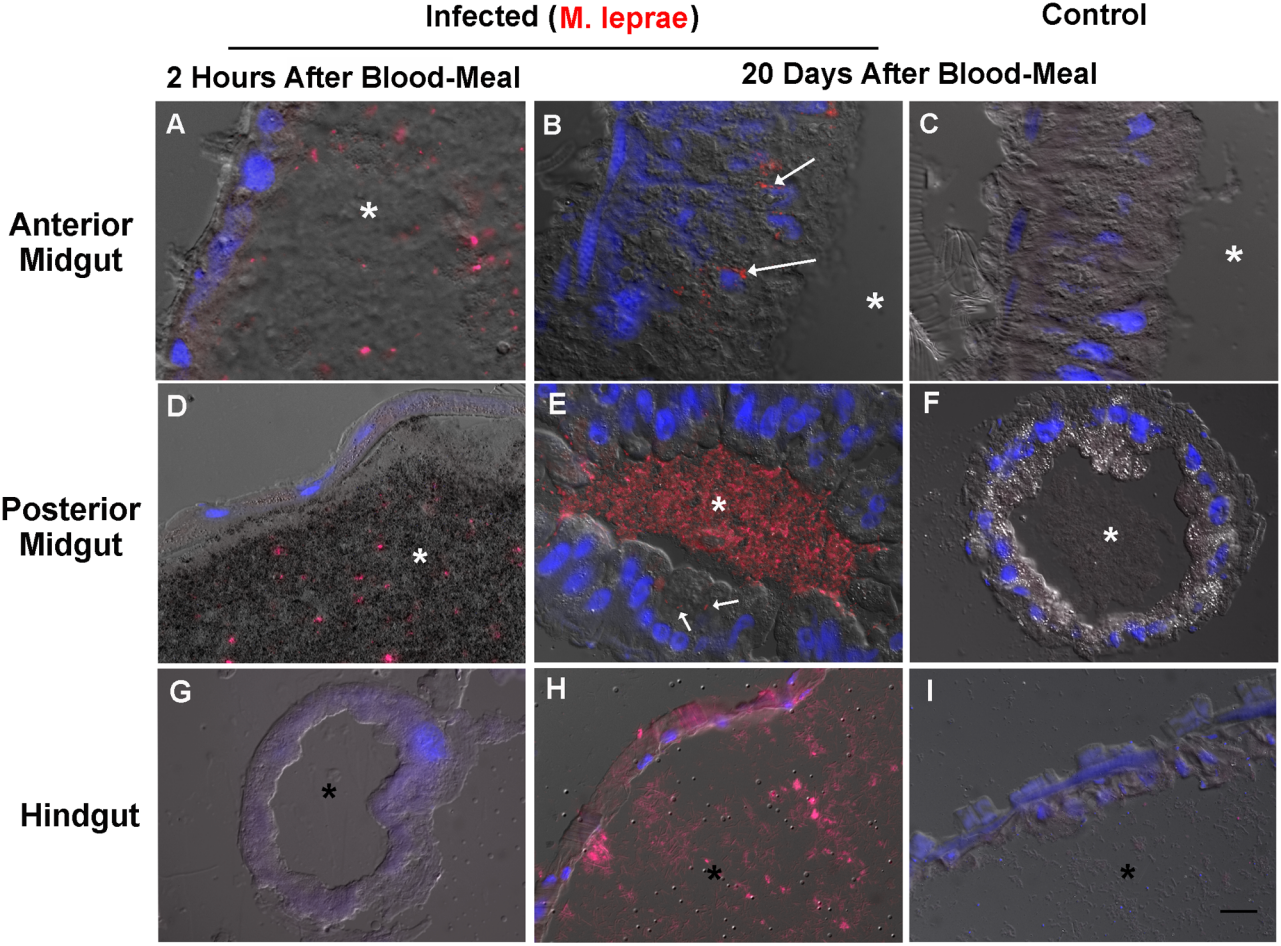
*M*. *leprae* rarely infects digestive epithelial cells of *Rhonius prolixus*. Immunohistofluorescence of tissue section from anterior midgut (A-C), posterior midgut (D-E) and hindgut (G-I) from infected and non-infected kissing bugs 2h and 20 days after infection. *M*. *leprae* was evidenced in red by IgG-LAM staining, and epithelial cells nuclei in blue by DAPI staining. Asterisks mark the luminal regions in all images, and arrows point to intracellular *M*. *leprae*. Images are representative of at least 15 fields, captured from six different insects, infected in three different experiments. Scale bar means 20μm.

In addition to PCR-based detection of live *M*. *leprae* cDNA, we performed immunolocalization of the parasite. Fig 3 demonstrates the immunohistofluorescence images from anterior midgut, posterior midgut and hindgut 2 hours and 20 days after the *M*. *leprae* infected or control blood meal. As we can see, a small number of mycobacteria could be observed after staining with anti-LAM IgG (red) inside anterior and posterior midgut epithelial cells (arrows) 20 days after infection. In fact, the majority of *M*. *leprae* signal was located in the luminal region of the posterior midgut and hindgut (asterisks), and virtually absent from the anterior midgut luminal region.

The posterior midgut epithelial cells, in contrast with the anterior one, are refractory to *M*. *leprae* infection, been scarcely colonized (arrows), maintaining the large majority of the bacillary load free in the luminal region (asterisk), on its way to the hindgut (Fig 3E). This indicates that the bacillus that did not get arrested at the anterior midgut epithelial cells, were dislocated within the luminal content to the hindgut (Fig 3H) through the posterior midgut (Fig 3E). Due to the huge number of bacteria in the luminal region, and the fragility and autofluorescence of the hindgut epithelia, we were not able to determine precisely the *M*. *leprae* location within epithelial cells in this tissue. Regarding the limitation in order to immunolocalize structures inside hindgut epithelia, *M*. *leprae* location is clearly predominantly luminal in this tissue, due to the proportional area of these two compartments.

**Fig 4:**
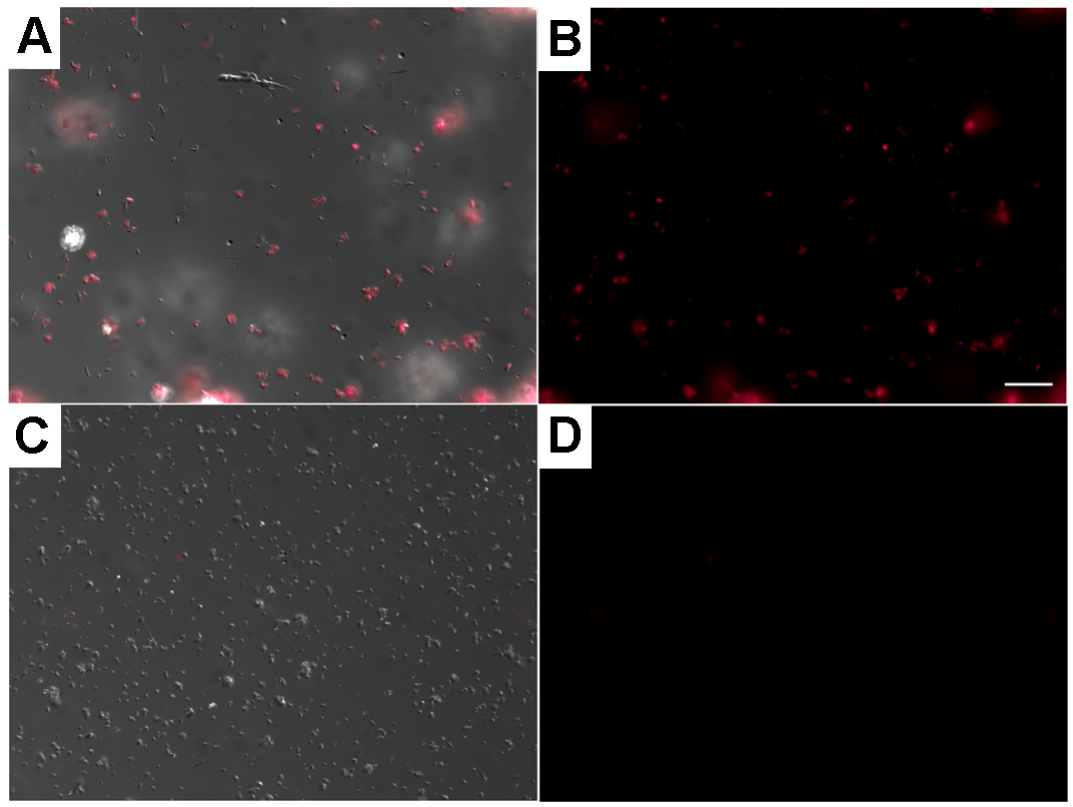
*M*. *leprae* reaches *Rhodnius prolixus* feces 20 days after infection. *Rhodnius prolixus* were allowed to feed rabbit blood containing (A-B) or not (C-D) 10^7^ PKH26-*M*. *leprae* / mL. During the next non-infected blood meal (20 days after infection), feces from groups of ten insects were collected in a sterile tube and analyzed by fluorescence microscopy (B and D). Stained *M*. *leprae* was identified only in the infected insects (A-B), allowing its counting. Images are representative of six pools of feces, where at least five fields were analyzed. Scale bar indicates 20μm.

In order to observe if the hindgut high titers of *M*. *leprae* observed by PCR are defecated during blood meal, six groups of 10 *R*. prolixus were fed with blood containing 10^7^/mL PKH-26 stained *M*. *leprae*. After 20 days, we offered a non-infected blood meal to these insects in sterilized tubes and collected their feces (Fig 4).

*M*. *leprae* amount in feces was evaluated by counting in a fluorescence microscope. We used this material for injecting six mice with 10^4^ *M*. *leprae* per BALB/c foot pad. After six months, animals were sacrificed, foot pads were macerated and acid-fast mycobacteria number estimated (Table 1). Among the animals, three demonstrated a tenfold increase and one animal presented one hundred times more bacteria than injected in the inoculum. Two animals did not demonstrate mycobacterial growth. After comparing bacillary counts of foot pads inoculated with *M*. *leprae* purified from *R*. *prolixus* feces with the positive and negative controls, where *M*. *leprae* viability is recognized to be higher than 90% and virtually absent respectively, we can see that four from six animals listed on Table 1 presented positive *M*. *leprae* infection. It is important to notice that *M*. *leprae* cell wall is a mycolic acid rich structure, able to persist intact for months mammal’s tissues [39], and for that reason we were able to count dead mycobacteria in negative controls. To conclude, we observed that *R*. *prolixus* infected by *M*. *leprae* through blood meal are able to maintain the initial inoculums alive, spreading to the environment feces containing infectious *M*. *leprae*.

**Table 1:** Numbers of *M*. *leprae* recovered from mice footpads after a six month infection with 10^4^ bacillus isolated from *Rhodnius prolixus* feces that received an infectious blood meal.

BalbC	1	2	3	4	5	6
*M*. *Leprae* from *R*. *prolixus* feces	1,3x10^6^	5,3x10^3^	3,5x10^5^	2x10^5^	1,1x10^5^	1,2x10^4^
Positive Control	1,6x10^6^	1,7x10^6^	3,4x10^6^	1,4x10^6^	1,7x10^6^	1,3x10^6^
Negative Control	1,0x10^4^	< 10^3^	1,2x10^4^	6,5x10^4^	6,9x10^4^	0,7x10^4^

Positive control was generated by 10^4^ Thai-53 *M*. *leprae* purified from nude mice foot pad inoculation. A negative control was generated by treating positive control animals with 10mg/Kg/week rifampicin by weekly gavage during five months, one month after inoculum with 10^4^ Thai-53 *M*. *leprae* purified from nude mice foot pad.

## Discussion

Until now, the impact of all possible aspects of transmission of *M*. *leprae* is unknown [40]. We investigated here the hypothesis that arthropods could contribute to the transmission of leprosy to vertebrates. This hypothesis was elaborated based on reports regarding the contribution of several insect species to the transmission of Buruli ulcer, a dermal infection caused by *M*. *ulcerans*, including predatory water bugs Hemipteras such as belostomatids and naucorids in Africa [41, 42] and mosquitos (Diptera) in Australia [43]. We therefore investigated if *M*. *leprae* could infect and maintain its viability inside the Dipteras *A*. *aegypti*, *C*. *quinquefasciatus* and the Hemiptera *R*. *prolixus*. The choice of these species was based on reports suggesting that *A*. *aegypti* [17, 18] and Culex sp. mosquitoes [20, 35] proboscis could be infected by *M*. *leprae* during the blood meal, mechanically transferring live mycobacteria during the next blood meal [21]. Our data that was focused in long term survival inside the digestive tract, indicates that *M*. *leprae* are not viable after 20 days inside mosquitoes digestive tract, which does not turn impossible its transmission through mechanical ways just after an incomplete blood meal in an infected mammal. Literature data estimates *M*. *leprae* viability inside mosquitoes through cell wall integrity accessed by microscopy for short periods of time from two [17] to 3 days [19]. On the other hand, longer observation periods in *A*. *aegypti* ranging from four [19] to nine days [17] did not demonstrate viable *M*. *leprae* in proboscis or posterior midguts, corroborating our data.

Different from what we observed for mosquitoes, we observed that the majority of the *M*. *leprae* inoculum was kept alive in the luminal region of the *R*. *prolixus* digestive tract, being epithelial cells scarcely infected by *M*. *leprae*. This resembles *T*. *cruzi* infection in *R*. *prolixus*, that also moves from the anterior midgut to hindgut [44]. Differently from the protozoan parasite, we did not observe significant proliferation of *M*. *leprae* in the insect gut, in spite of its capacity to maintain viability during prolonged periods. This discrepancy observed between mosquitoes and kissing bug could be attributed to the differences between their digestive tract and intestinal microflora control mechanisms. Both groups of insects are able to generate oxygen and nitrogen reactive intermediates through prophenoloxidase system in their haemolymph [45, 46]. But recently a new microflora control mechanism was described on *Aedes aegypti* digestive tract. This mechanism involves the formation of reactive intermediates of oxygen by epithelial cells mitochondria, which are responsible for drastically reduce the bacterial load in midgut luminal region 56h after the blood meal [47]. This mechanism could be involved in the complete abolishment of *M*. *leprae* viability during blood meal digestion in both mosquitoes analyzed on the present work.

Due to a drastic reduction of its genome, suffered during its adaptation to live inside a eukaryotic cell, *M*. *leprae* presents 1116 pseudogenes in contrast with 6 in *M*. *tuberculosis* [48]. Based on this, the observation that the majority of living *M*. *leprae* maintained in the kissing bug midgut is located in the extracellular intestinal lumen contrasts with the usual idea that *M*. *leprae* is an obligatory intracellular pathogen [40]. This particular environment represented by *R*. *prolixus* digestive tract: microaerobic presenting high amounts of heme-iron and blood-proteins, pH 5,0 and 28°C, maintained *M*. *leprae* viability almost intact for 20 days. This is better than the viability observed in Middlebrook 7H12 at 4°C in the same period of time, a condition recognized as the best alternative to maintain extracellular *M*. *leprae* viability [31].

In our model we applied 10^7^ *M*. *leprae* / blood mL as initial inoculum in order to successfully trace *M*. *leprae* viability in all intestinal compartments. Since an adult kissing bug can ingest 250 μl of blood and mosquitoes 2 μl, this represents an initial inoculum around 2.5x10^6^ and 2x10^4^ bacteria per insect respectively. Although this high number of circulating bacteria is improbable to occur in humans, it is possible in infected wild armadillos.

In order to control leprosy efficiently, observing a strong reduction in the number of new cases in the endemic countries, we need to identify other transmission factors beyond multibacillary patients. Multibacillary patients are considered as the primary source of infection of leprosy. The introduction of WHO defined chemotherapy in 1981 reduced in 90% the prevalence of the disease with a reduction in the number of new cases of 50% since then [1, 3]. It therefore seems that treatment of multibacillary patients has no impact in the control of leprosy transmission, since the number of new cases around the world remained at similar levels during the last two decades [49]. Leprosy was virtually eradicated from Norway one century before the development of an efficient treatment against the disease [50] by means of industrialization, migration of the population from the countryside to the cities and deforestation of innumerous habitats of insects and small mammals [51–53]. New evidences point that other factors contribute to the maintenance of the disease among humans, such as water and soil contaminated with *M*. *leprae* infected amoebas [54].

The mode of Leprosy transmission by *Rhodnius prolixus* kissing bug hypothesized here is analogous to that described for the contribution of insect species to the transmission of Buruli ulcer, a dermal infection caused by *M*. *ulcerans*, for which transmission by predatory water bugs Hemipteras, such as belostomatids and naucorids, has been reported in Africa [41, 42] and by mosquitos (Diptera) in Australia [43]. The hemipterans present potential as leprosy vectors, since kissing bugs usually feed on nine-band armadillos, the most acceptable wild reservoir of leprosy [12, 24]. In fact, about 1% of nine-band armadillos population in the south of US is co-infected by *M*. *leprae* and *T*. *cruzi* [23]. Although *M*. *leprae* persistence inside *R*. *prolixus*, or even in free-living amoebae [54], is not a proof of involvement of these organisms in leprosy transmission, we hypothesize that kissing bugs, well distributed in Americas, Asia and Africa could be infected by *M*. *leprae* during blood meal in natural reservoirs such as armadillos in Americas. These infected kissing bugs could develop a role in the endemic leprosy observed in the Brazilian agricultural frontier, where we can find high incidence of leprosy, armadillos and triatomines [24].

Our data indicate that kissing bugs are able to defecate infectious *M*. *leprae* after being infected through a blood meal. The incidence of kissing bugs infected by *M*. *leprae* in highly endemic areas is currently under investigation by our group, through detection of the presence of live *M*. *leprae* in kissing bugs captured from leprosy patients domiciliary and peridomiciliary areas in Brazilian countryside.

## Supporting Information

S1 Fig Inactivated *M*. *leprae* was not able to sustained detectable levels of 16SrRNA after 20 days inside digestive tract. Freeze-thaw inactivated *M*. *leprae* 16Sr RNA persistence at the different digestive compartments of adult *Rhodnius prolixus*: anterior midgut (spheres), posterior midgut (squares) and hindgut (triangles), just after blood meal (2h) and after total blood meal digestion (20 days). Scatter plot showing mean and SEM of four independent experiments, each point represent five insects group. *** means p < 0.001. Controls did not present amplification of the targets.
(TIF)
