# Supplementary figures and images for: Experimental Infection of Rhodnius prolixus (Hemiptera, Triatominae) with Mycobacterium leprae Indicates Potential for Leprosy Transmission
Source: PLoS One. 2016 May 20;11(5):e0156037. doi: 10.1371/journal.pone.0156037 (PMC4874629; doi:10.1371/journal.pone.0156037)

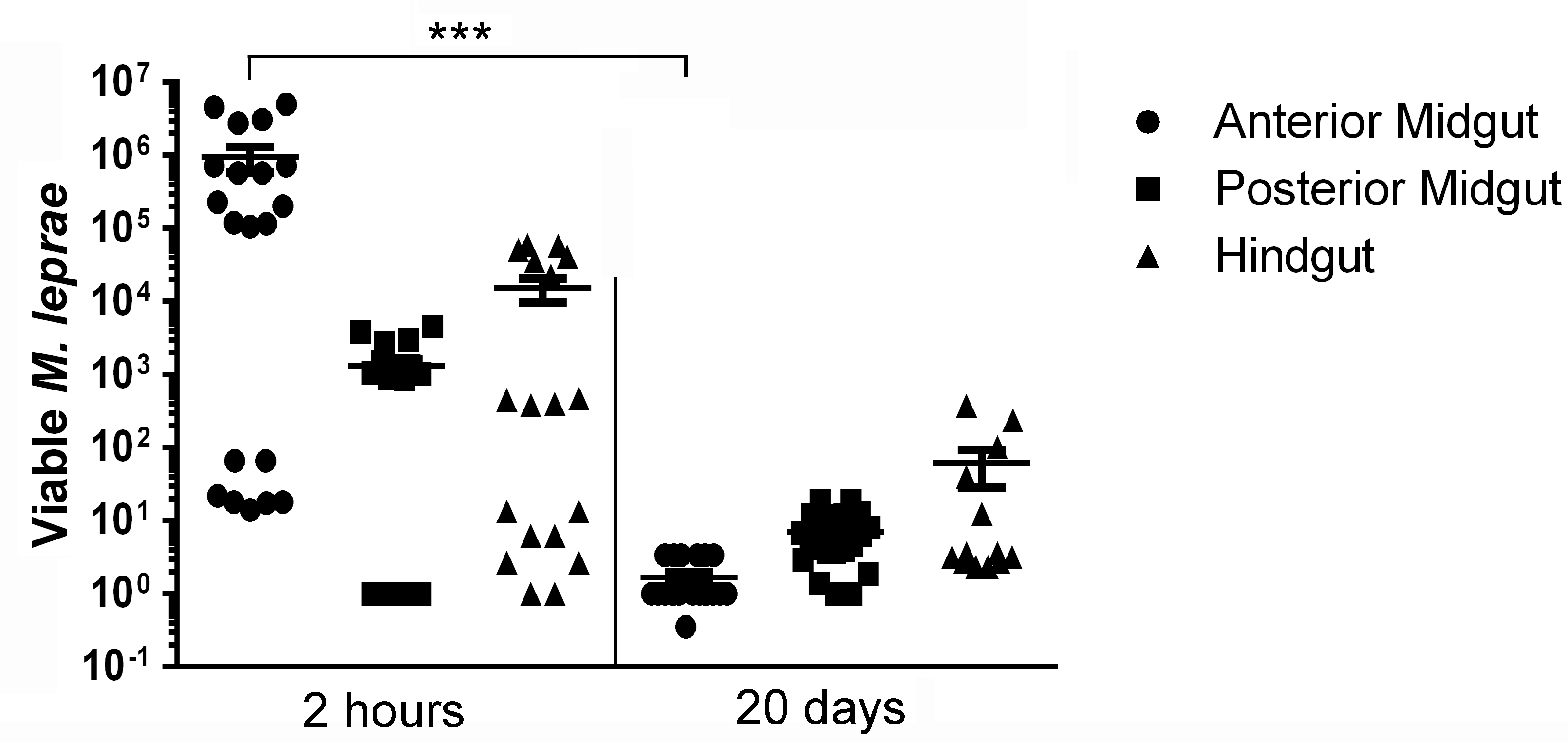

Supplement: S1 Fig — Freeze-thaw inactivated M. leprae 16Sr RNA persistence at the different digestive compartments of adult Rhodnius prolixus: anterior midgut (spheres), posterior midgut (squares) and hindgut (triangles), just after blood meal (2h) and after total blood meal digestion (20 days). Scatter plot showing mean and SEM of four independent experiments, each point represent five insects group. *** means p < 0.001. Controls did not present amplification of the targets. (TIF) [file pone.0156037.s001.TIF]
